# Five-year-outcome of new-onset perioperative atrial fibrillation after left atrial appendage amputation concomitant with cardiac surgery

**DOI:** 10.1007/s00392-023-02255-8

**Published:** 2023-07-10

**Authors:** Mustafa Gerçek, Jochen Börgermann, Jan Gummert, Muhammed Gerçek

**Affiliations:** 1 Heart Center Duisburg, Clinic for Cardiac Surgery and Pediatric Cardiac Surgery, Gerrickstraße 21, 47137 Duisburg, Germany; 2 Herz- und Diabeteszentrum NRW, Clinic for Thoracic and Cardiovascular Surgery, Georgstraße 11, 32545 Bad Oeynhausen, Germany; 3 Herz- und Diabeteszentrum NRW, Clinic for General and Interventional Cardiology/Angiology, Georgstraße 11, 32545 Bad Oeynhausen, Germany

**Keywords:** POAF, LAA amputation, Cardiac surgery, OPCAB, Off-pump

## Abstract

**Background:**

Recent data demonstrated the benefit of left atrial appendage (LAA)-amputation in patients with atrial fibrillation (AF). However, the long-term impact of LAA-amputation for patients with new-onset perioperative atrial fibrillation (POAF) is still unknown.

**Methods:**

Patients with no history of AF undergoing coronary artery bypass grafting by off-pump technique (OPCAB) between 2014 and 2016 were retrospectively examined. Cohorts were divided by the concomitant execution of LAA-amputation. Propensity score (PS) matching was applied by all available baseline characteristics. The composite of all-cause mortality, stroke and rehospitalization in patients with POAF and patients maintaining sinus rhythm posed as the primary endpoint.

**Results:**

A total of 1522 patients were enrolled, of whom 1208 and 243 were included in the control and the LAA-amputation group, respectively and were matched to 243 patients in each group. In total, patients with POAF without LAA-amputation showed a significantly higher rate of the composite endpoint (17.3% vs 32.1%, p = 0.007). However, patients with LAA-amputation showed no significant difference in the composite endpoint (23.2% vs 26.7%, p = 0.57). The significantly higher occurrence of the composite endpoint was driven by all-cause mortality (p = 0.005) and rehospitalization (p = 0.029). Subgroup analysis revealed a CHA_2_DS_2_-VASc-score of ≥ 3 to be associated with the high rate of the primary endpoint (p = 0.004).

**Conclusion:**

POAF is associated with a higher rate of the combined endpoint of all-cause mortality, stroke and rehospitalization. The composite endpoint in patients with LAA-amputation concomitant with OPCAB surgery developing new-onset POAF in a 5-year follow-up was not increased compared to a control cohort maintaining sinus rhythm.

**Graphical abstract:**

Five-year outcome of patients with POAF and LAA-amputation; *95% CI*, 95% confidence interval, *CPR,* cardiopulmonary resuscitation, *ECLS,* extracorporeal life support, *HR,* hazard ratio, *IABP*, intra-aortic balloon pump, *LAA*, left atrial appendage, *OPCAB*, off-pump coronary artery bypass grafting, *PAPs*, systolic pulmonary artery pressure, *SR*, sinus rhythm, *VT,* ventricular tachycardia.

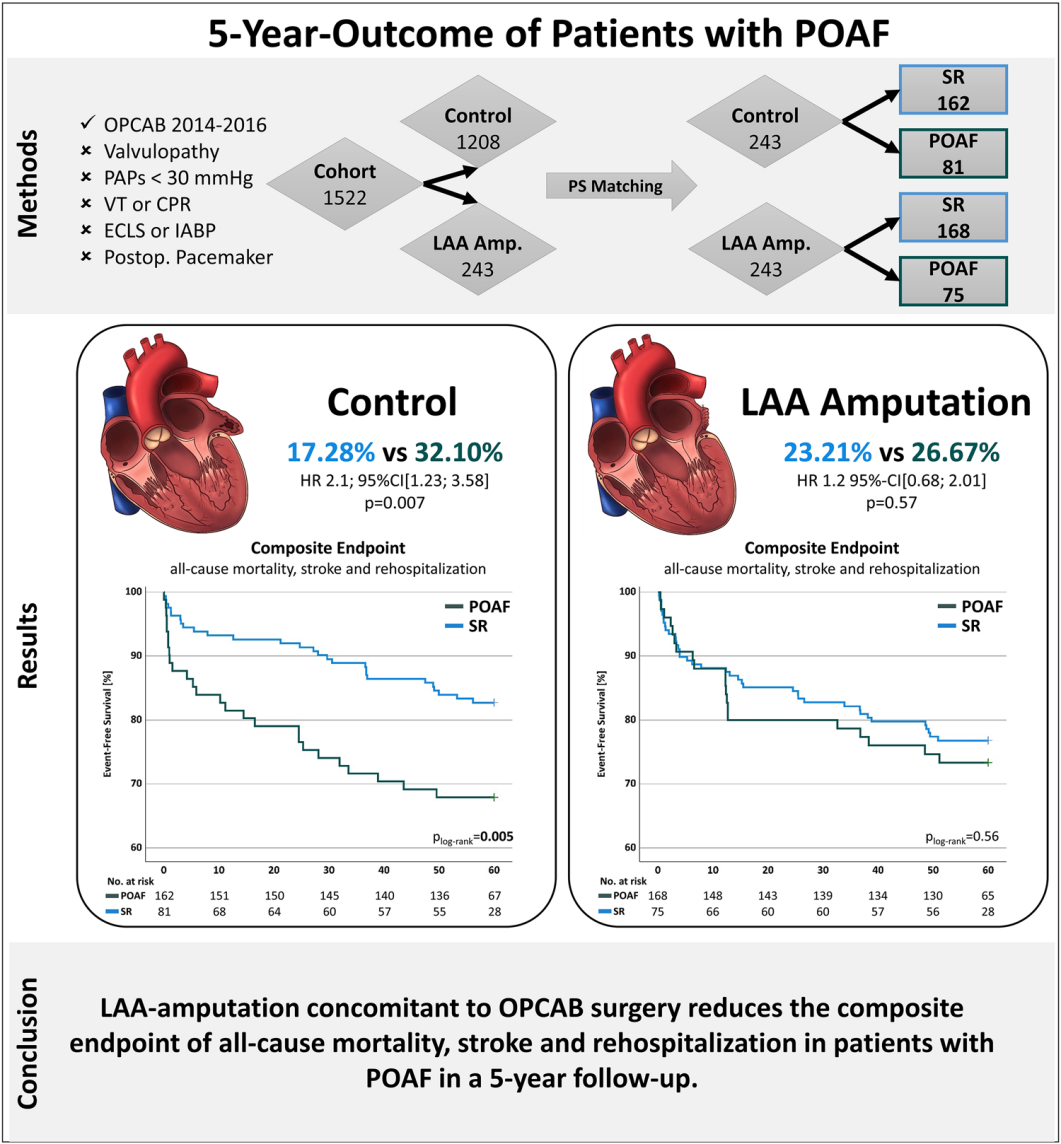

**Supplementary Information:**

The online version contains supplementary material available at 10.1007/s00392-023-02255-8.

## Introduction

Perioperative atrial fibrillation (POAF) is a common complication in cardiac surgery, associated with a high rate of long-term atrial fibrillation (AF), decreased cognitive and renal function, as well as an elevated use of medical resources such as extended hospitalizations and the need of intensive care and also an increased mortality rate [1–3]. The occurrence rate of POAF is varying between 20 and 50% [4, 5]. Therefore, the current guidelines recommend restoring sinus rhythm by antiarrhythmic medication, as well as electrical cardioversion, and reducing the risk of thromboembolic stroke by anticoagulation [6].

**Fig. 1:**
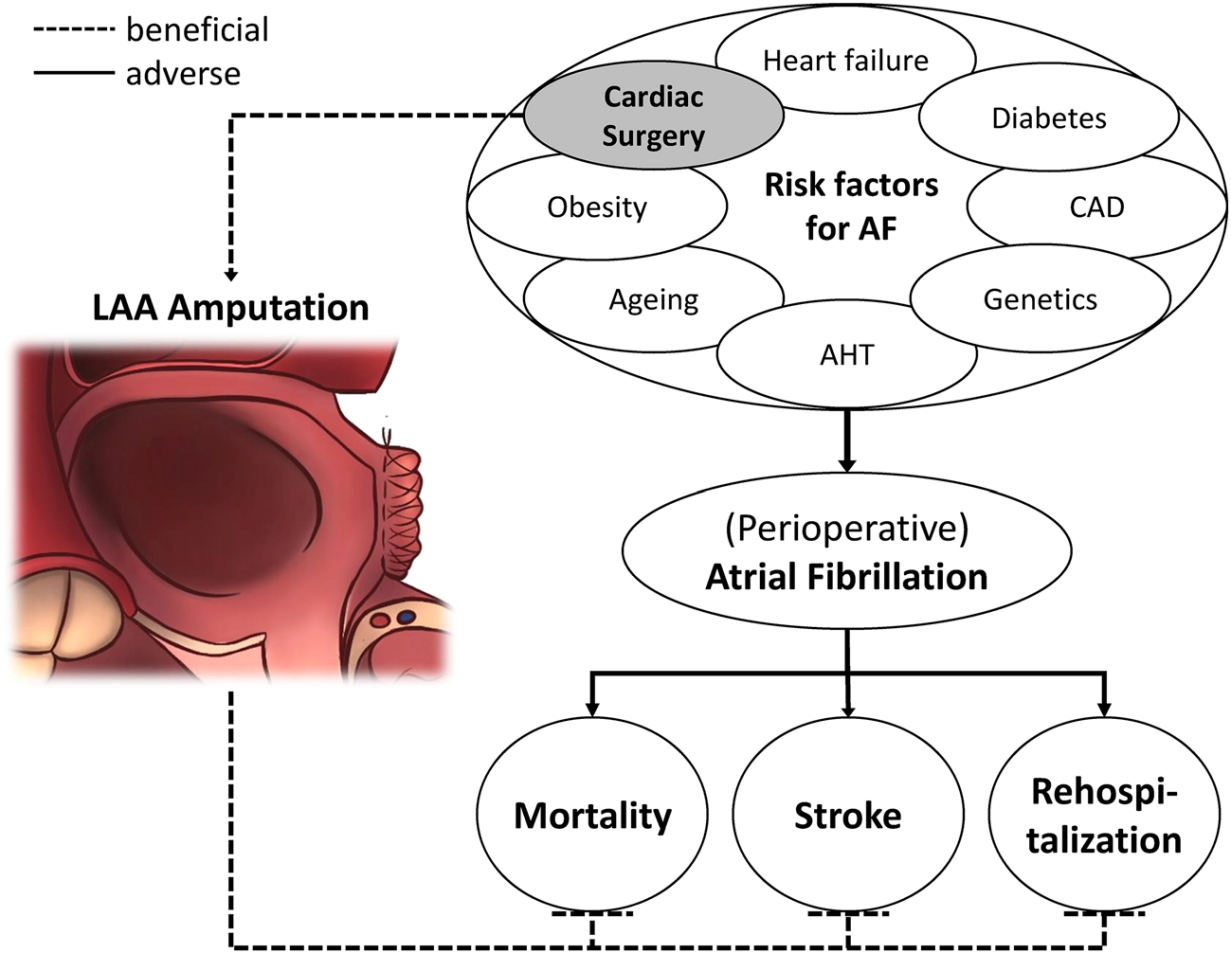
Hypothesis of stroke prevention by LAA-amputation; *CAD* coronary artery disease, *LAA* left atrial appendage

Once POAF occurs, the range of complications is similar to none-surgery related atrial fibrillation. For that reason, surgery-related AF, along with several other factors predisposing to develop AF such as age, genetic predisposition, diabetes mellitus, arterial hypertension, or obesity [7–9], should be considered as a relevant comorbidity (Fig. 1).

The baseline strategy and gold standard for stroke prevention in persistent AF is medical anticoagulation [6]. Since the left atrial appendage (LAA) is known to be the major origin of stroke caused by thrombi [10], the exclusion of the left atrial appendage by intervention or surgery (concomitant with cardiac surgery) has become a field of rising interest [10].

In fact, the LAA is easy to access during cardiac surgery by median sternotomy or anterolateral thoracotomy, leading to develop and establish techniques to exclude the LAA through ligation or amputation concomitant with e.g. coronary or valvular cardiac surgery [11, 12]. The previous hesitant recommendations to perform LAA-amputation have been strengthened by increasing evidence of stroke prevention through LAA-amputation in patients with AF, with the most famous to mention LAAOS III trial [13].

However, stroke prevention through LAA-amputation has also been observed in patients with sinus rhythm and a high CHA_2_DS_2_-VASc-score [14]. Thus, the prophylactic impact of LAA-amputation on stroke, all-cause mortality, and rehospitalization might be most effective in POAF patients. However, a dedicated analysis with long-term follow-up in POAF patients undergoing LAA-amputation has yet to be performed.

## Patients and methods

### Ethical statement

Approval including patient consent waiver was obtained by the local ethics committee of the Ruhr University Bochum (No: 2020-688_1; Date: 12.08.2022). Every patient received a thoroughly dedicated patient information concerning the LAA-amputation. The investigation was performed in accordance with the STrengthening the Reporting of OBservational studies in Epidemiology (STROBE) statement (www.strobe-statement.org). Furthermore, the study was conducted in accordance with the ethical standards laid down in the 1964 Declaration of Helsinki and its later amendments.

### Patient recruitment and follow up

All patients undergoing isolated coronary artery bypass grafting in off-pump technique (OPCAB) between January 2014 and December 2016 in our department (Herz- und Diabeteszentrum NRW, Bad Oeynhausen, Germany) were retrospectively selected in a single-center assessment. Preoperative exclusion criteria were a history of AF, valvulopathy, and pulmonary arterial hypertension. Perioperative exclusion criteria were the need of a permanent pacemaker, extracorporeal life support, intraaortic balloon pump, ventricular tachycardia, resuscitation, or defibrillation. A detailed description of the patient recruitment strategy was given before [15].

### Surgical technique

The surgical procedure was performed utilizing the off-pump technique through median sternotomy, and well-established grafts such as the left and/or right internal mammary artery, radial artery, and saphenous vein were employed. After pericardial exposure the accessibility of the LAA was judged by the surgeon. If the LAA was deemed accessible and suitable for amputation, the procedure was completed by ligation, LAA resection, and double continuous suture. Transesophageal echocardiography was conducted both before and after LAA amputation to detect any pre-existing thrombus formation or residual appendage post-amputation.

### Anticoagulation

All patients were initially treated with dual antiplatelet therapy consisting of acetylsalicylic acid 100 mg and Clopidogrel 75 mg (a P2Y12 inhibitor). The dual antiplatelet regimen was recommended for a duration of 6 months, after which a transition to mono antiplatelet therapy with acetylsalicylic acid 100 mg was advised. If POAF occurred, a switch of the dual antiplatelet therapy to a combination of single antiplatelet (acetylsalicylic acid 100 mg) and oral anticoagulation strategy (Phenprocoumon) for at least 3 months was recommended. Further follow-up of the anticoagulation therapy was not part of the presented data.

### POAF

**Fig. 2:**
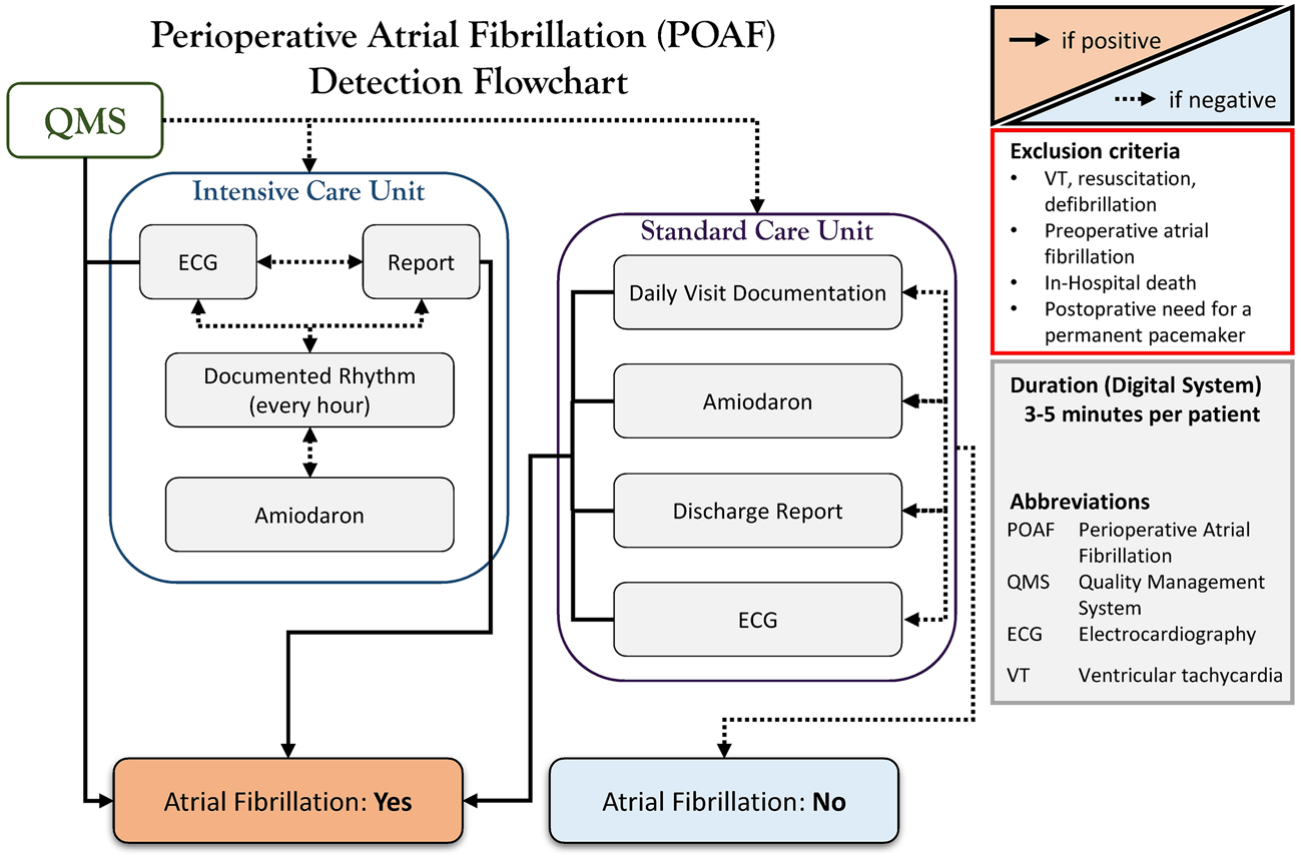
Browsing flowchart for detection of new-onset perioperative atrial fibrillation

To retrospectively detect the occurrence of POAF, a detailed browsing flowchart was developed, as described before [15]. In brief, prehospital reports, in-hospital transfer and discharge reports, any custodial and visit documentation in the data management systems of the intensive (COPRA, COPRA System GmbH; Germany) and standard care (ORBIS, Dedalus HealthCare, Germany) units were analyzed for any signs of arrhythmia. Furthermore, patient data was inspected for the use of antiarrhythmic medication, whereas a rhythm control was achieved by per standard used postoperative telemetry until discharge. ECG was performed at least four times for each patient (on admission, immediately after surgical intervention, first postoperative day, and before discharge). A flowchart summarizing the POAF detection protocol is provided in Fig. 2.

### Outcome

The primary outcome was defined as a composite endpoint of all-cause mortality, stroke and rehospitalization. Secondary endpoints were defined as the isolated parameters of the composite endpoint. The endpoint stroke was defined as either the confirmation of stroke through cerebral imaging (computed tomography or MRI) or the presence of unequivocal neurological impairment, such as hemiplegia. Additionally, instances of stroke confirmed by a neurologist reported to our department during the follow-up period were also included in the definition. The endpoints were assessed by using four sources of information: a review of our medical records; an annual, standardized form (post-discharge) completed by the patients themselves, as well as by their out-patient care physician, and annual consultation with the respective registration office in case of missing post-discharge forms. Follow-up data was then assessed between the date of surgery (2014–2016) and December 2021. Patients were censored at their last follow-up.

To assess subgroup differences, subgroup analyses of older patients (age > 70) and patients with an increased stroke risk (CHA_2_DS_2_-VASc-score ≥ 3) were performed. All analyses were done in the total cohort, the LAA-amputation cohort, and the control cohort.

### Statistical analysis

Statistical analysis was performed using the SPSS-Software (Version 28, IBM, New York, NY, USA) and R (Version 4.2.2, R Core Team, Vienna, Austria). Categorical variables are given as absolute and relative frequencies, continuous variables as means with standard deviations. Since the surgical technique was chosen in a nonrandomized fashion, we used 1:1 PS matching by 20 baseline characteristics using nearest neighbor matching with a caliper of 0.2 (Table 1), which was described in detail before[15], resulting in groups of 243 patients with and without LAA-amputation. To assess the patient outcome, patients were divided into an SR group and a POAF group. To conclude, the balance of baseline covariates was assessed by computing the z-difference (balance achieved, if ≤ |± 1.96|) and for categorical variables by additionally computing the standardized mean difference (balance achieved if < 0.1) [16, 17].

**Table 1:** Baseline characteristics of the PS matched cohorts

Variable	PS matched cohorts			
	LAA-amputation		z-difference	Std. mean difference
	No (n = 243)	Yes (n = 243)		
NYHA (%)			0.24	–
NYHA I	75 (29.8)	68 (27.0)		
NYHA II	103 (40.9)	113 (44.8)		
NYHA III	71 (28.2)	68 (27.0)		
NYHA IV	3 (1.2)	3 (1.2)		
Ejection fraction (SD)	58.2 (9.1)	57.6 (9.9)	− 0.68	0.06
DVT (%)	4 (1.6)	5 (2.0)	0.34	0.03
Beta-blocker (%)	179 (71.0)	170 (67.5)	− 0.87	0.08
Calcium antagonist (%)	64 (25.4)	66 (26.2)	0.20	0.02
EuroSCORE2 (IQR)	1.2 (1.1)	1.3 (1.2)	0.28	0.03
CHA_2_DS_2_-VASc-score (SD)	3.1 (1.4)	3.1 (1.3)	0.13	0.01
Age (SD)	69.6 (8.1)	69.1 (7.6)	− 0.70	0.06
BMI (SD)	29.1 (4.3)	29.1 (4.3)	− 0.05	0.01
TSH (µU/ml) (IQR)	1.1 (1.0)	1.1 (1.0)	0.55	0.05
ASA classification (%)			0.30	–
Normal healthy patient	13 (5.2)	13 (5.2)		
Patient with mild systemic disease	21 (8.3)	20 (7.9)		
Patient with severe systemic disease	207 (82.1)	206 (81.7)		
Patient with severe systemic disease that is a constant threat to life	11 (4.4)	13 (5.2)		
CCS (%)			− 0.39	–
No	70 (27.8)	71 (28.2)		
CCS I	80 (31.7)	85 (33.7)		
CCS II	58 (23.0)	56 (22.2)		
CCS III	41 (16.3)	34 (13.5)		
CCS IV	3 (1.2)	6 (2.4)		
Female (%)	48 (19.0)	54 (21.4)	0.67	0.06
Myocardial infarction (%)			0.29	–
Longer than 90 days	32 (12.7)	31 (12.3)		
21–90 days	6 (2.4)	6 (2.4)		
7–21 days	8 (3.2)	7 (2.8)		
48 h–7 days	2 (0.8)	3 (1.2)		
24–48 h	2 (0.8)	4 (1.6)		
6–24 h	1 (0.4)	1 (0.4)		
0–6 h	0 (0.0)	1 (0.4)		
Arterial hypertension (%)	226 (89.7)	223 (88.5)	− 0.43	0.04
Diabetes mellitus type 2 (%)	100 (39.7)	105 (41.7)	0.45	0.04
Pulmonary disease (%)	16 (6.3)	20 (7.9)	0.70	0.06
Hyperlipidemia (%)	239 (94.8)	234 (92.9)	− 0.94	0.08
Smoking (%)	93 (36.9)	101 (40.1)	0.73	0.07
Not used for PSM				
Coronary artery disease (%)	2.8 ± 0.5	2.9 ± 0.3	0.13	
1-CAD	9 (3.7)	0 (0)		
2-CAD	36 (14.8)	27 (11.1)		
3-CAD	198 (81.5)	216 (88.9)		
Operation time (min)	196.4 ± 46.5	199.5 ± 37.8	0.36	

*ASA* American Society of Anesthesiologists, *BMI* body mass index, *CAD* coronary artery disease, *DVT* deep vein thrombosis, *IQR* interquartile range, *NYHA* New York Heart Association, *PSM* propensity score matching, *TSH* thyroid stimulating hormone

A time-to-event analysis was performed regarding the primary composite endpoint and the secondary endpoints with the use of Kaplan–Meier survival curves, stratified log-rank testing, and a stratified Cox proportional-hazards model. Categorical baseline variables were compared with the use of the Chi-square test or Fisher’s exact test.

Parameter estimates are given with their hazard ratio, 95% confidence interval (95% CI) and the corresponding p value. p values < 0.05 were considered statistically significant.

## Results

### Cohort

After the exclusion of 59 patients (4.7%) in the control group and 12 patients (4.7%) in the LAA-amputation group due to perioperative exclusion criteria, a total of 1208 and 243 patients were included, respectively. These were paired via PS matching to 243 patients in each group, achieving a standardized mean difference of < 0.1 and a z-difference of ≤ |± 1.96|. Twenty (8.2%) of the patients in the LAA group were included despite showing a CHA_2_DS_2_VASc-score of 1, due to smoke/thrombus formation in perioperative transesophageal echocardiography. The mean age of the patients was 69.6 (8.1) and 69.1 (7.6) years in the control and LAA-amputation group, respectively, while 19.0% and 21.4% were female. POAF after OPCAB was observed in 31.2% of LAA patients compared to 33.3% in the control group (HR 0.94; 95% CI [0.62; 1.41], p = 0.75) [15]. The baseline characteristics of the matched cohorts are illustrated in Table 1. Baseline characteristics divided by the development of POAF are provided in supplementary table 1.

### POAF in the total cohort

**Fig. 3:**
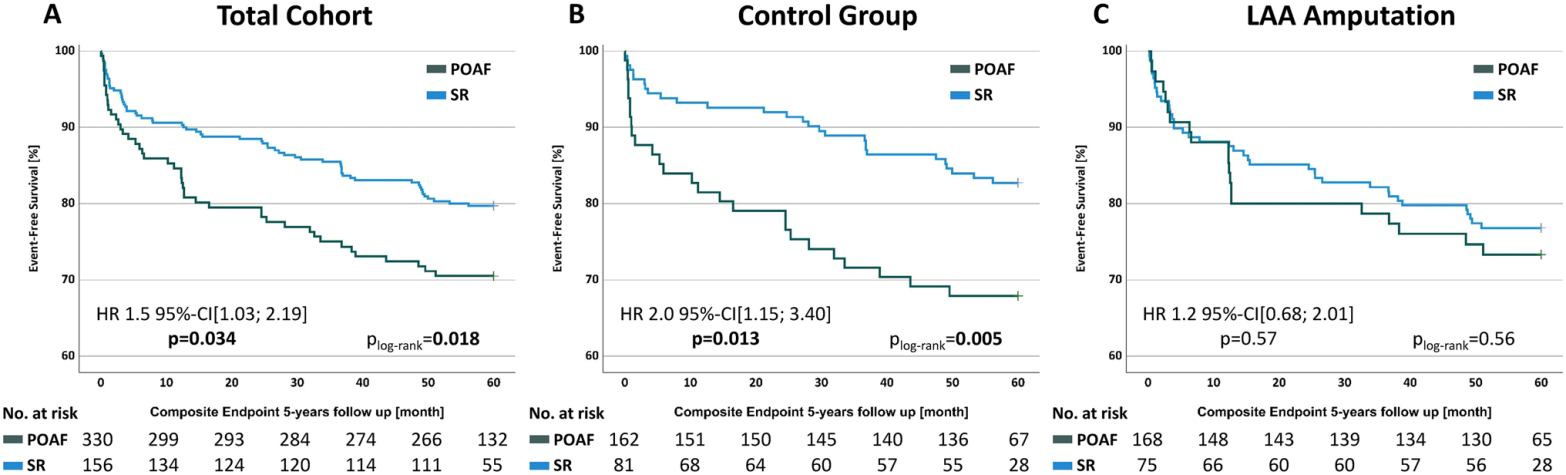
Survival analysis of the primary composite endpoint; *HR* hazard ratio, *POAF* perioperative atrial fibrillation, *SR* sinus rhythm

Five-year results revealed a significantly higher frequency of the composite endpoint in the POAF group with 20.3% vs. 29.5% (HR 1.5 95% CI [1.03; 2.19] p = 0.034) and a higher all-cause mortality 11.2% vs. 20.5% (HR 1.8 95% CI [1.14; 2.95] p = 0.012) (Table 2, Fig. 3A).

**Table 2:** Primary composite and single endpoints of patients with perioperative maintenance of sinus rhythm and perioperative atrial fibrillation in the total cohort (with and without LAA-amputation)

Total cohort (n = 486)	SR [n (%)]	POAF [n (%)]	HR [95% CI]	p value
	330	156		
Composite endpoint	67 (20.3)	46 (29.5)	1.5 [1.03; 2.19]	**0.034**
All-cause mortality	37 (11.2)	32 (20.5)	1.8 [1.14; 2.95]	**0.012**
Stroke	14 (4.2)	10 (6.4)	1.5 [0.68; 3.47]	0.30
Rehospitalization	19 (5.8)	15 (9.6)	1.8 [0.92; 3.61]	0.08

*HR* hazard ratio, *POAF* perioperative atrial fibrillation, *SR* sinus rhythm

Bold value indicates *p*-values < 0.05 were considered statistically significant

### Subgroup analysis in the total cohort

In the subgroup analysis of the total cohort regarding elderly patients (age > 70), a tendency towards a higher occurrence of the composite endpoint was observed [22.2% vs 31.1 (HR 1.5 95% CI [0.94; 2.42]) p = 0.09] driven by a significantly higher rate of all-cause mortality [14.6% vs 25.2% (HR 1.8 95% CI [1.06; 3.18]) p = 0.031] and a higher susceptibility for rehospitalization [2.9 vs 7.8 (HR 2.8 95% CI [0.91; 8.55]) p = 0.07], whereas the rate of stroke did not differ (p = 0.77). In younger patients (age ≤ 70) neither the composite [18.2% vs 26.4% (HR 1.6 95% CI [0.81; 3.00]) p = 0.18] nor the single endpoints showed a significant difference.

Regarding the comorbidity dependent stroke risk, the subgroup with a CHA_2_DS_2_VASc-score < 3 did not show a notable difference of the endpoints [composite endpoint 12.4% vs 14.6% (HR 1.2 95% CI [0.49; 2.78] p = 0.74)]. However, the subgroup with a score of ≥ 3 presented a substantially higher occurrence of the composite endpoint [24.9% vs 37.6% (HR 1.7 95% CI [1.09; 2.53] p = 0.018)] and the single endpoints all-cause mortality [HR 1.7 95% CI [1.03; 2.88] p = 0.039)] and rehospitalization [HR 2.5 95% CI [1.14; 5.5] p = 0.022)], whereas the outcome of stroke did not differ (p = 0.13). Detailed results of the subgroups in the total cohort are presented in Table 3.

**Table 3:** Subgroup analysis of older age (> 70 years) and high stroke risk (CHA_2_DS_2_-VASc-Socre ≥ 3 in all patients

	Total cohort			
	SR [n (%)]	POAF [n (%)]	HR [95% CI]	p value
Age				
Age ≤ 70	159	53		
Composite endpoint	29 (18.2)	14 (26.4)	1.6 [0.81; 3.00]	0.18
All-cause mortality	12 (7.6)	6 (11.3)	1.5 [0.55; 4.07]	0.43
Stroke	5 (3.1)	4 (7.6)	2.5 [0.65; 9.53]	0.19
Rehospitalization	14 (8.8)	7 (13.2)	1.7 [0.68; 4.39]	0.25
Age > 70	171	103		
Composite endpoint	38 (22.2)	32 (31.1)	1.5 [0.94; 2.42]	0.09
All-cause mortality	25 (14.6)	26 (25.2)	1.8 [1.06; 3.18]	**0.031**
Stroke	9 (5.3)	6 (5.8)	1.2 [0.42; 3.29]	0.77
Rehospitalization	5 (2.9)	8 (7.8)	2.8 [0.91; 8.55]	0.07
Stroke risk				
CHA_2_DS_2_-VASc-score < 3	121	55		
Composite endpoint	15 (12.4)	8 (14.6)	1.2 [0.49; 2.78]	0.74
All-cause mortality	4 (3.3)	6 (10.9)	3.2 [0.89; 11.39]	0.08
Stroke	4 (3.3)	1 (1.8)	0.6 [0.06; 4.95]	0.59
Rehospitalization	7 (5.8)	2 (3.6)	0.7 [0.14; 3.41]	0.65
CHA_2_DS_2_-VASc-score ≥ 3	209	101		
Composite endpoint	52 (24.9)	38 (37.6)	1.7 [1.09; 2.53]	**0.018**
All-cause mortality	33 (15.8)	26 (25.7)	1.7 [1.03; 2.88]	**0.039**
Stroke	10 (4.8)	9 (8.9)	2.0 [0.81; 4.94]	0.13
Rehospitalization	12 (5.7)	13 (12.9)	2.5 [1.14; 5.50]	**0.022**

*HR,* hazard ratio, *POAF,* perioperative atrial fibrillation, *SR*, sinus rhythm

Bold value indicates *p*-values < 0.05 were considered statistically significant

### POAF in the LAA-amputation and control group

Among the control group, patients with POAF showed a worse outcome with a considerably higher frequency of the composite endpoint [17.3% vs 32.1% (HR 2.0 95% CI [1.15; 3.40] p = 0.013)] and the single endpoints all-cause mortality [8.0% vs 21.0% (HR 2.6 95% CI [1.26; 5.39] p = 0.010)] and rehospitalization [4.3% vs 11.1% (HR 2.8 95% CI [1.04; 7.69] p = 0.043)].

However, patients with LAA-amputation and POAF did not show a significantly higher rate of the composite and single endpoints [composite endpoint 23.2% vs 26.7% (HR 1.2 95% CI [0.68; 2.01] p = 0.57)]. Detailed results and survival curves are provided in Table 4 and Fig. 3.

**Table 4:** Five-year results of the primary composite and single endpoints in patients with perioperative maintenance of sinus rhythm and perioperative atrial fibrillation in the LAA-amputation group and the control group

Variable	SR [n (%)]	POAF [n (%)]	HR [95% CI]	p value
Control				
	162	81		
Composite endpoint	28 (17.3)	26 (32.1)	2.0 [1.15; 3.40]	**0.013**
All-cause mortality	13 (8.0)	17 (21.0)	2.6 [1.26; 5.39]	**0.010**
Stroke	10 (6.2)	7 (8.6)	1.5 [0.57; 4.01]	0.41
Rehospitalization	7 (4.3)	9 (11.1)	2.8 [1.04; 7.69]	**0.043**
LAA amp				
	168	75		
Composite endpoint	39 (23.2)	20 (26.7)	1.2 [0.68; 2.01]	0.57
All-cause mortality	24 (14.3)	15 (20.0)	1.4 [0.76; 2.75]	0.26
Stroke	4 (2.4)	3 (4.0)	1.7 [0.39; 7.70]	0.48
Rehospitalization	12 (7.1)	6 (8.0)	1.2 [0.44; 3.14]	0.74

*HR* hazard ratio, *POAF* perioperative atrial fibrillation, *SR* sinus rhythm

Bold value indicates *p*-values < 0.05 were considered statistically significant

### Subgroup analysis in the LAA-amputation and control group

Regarding the subgroup analysis in the LAA-amputation and control group, no subgroup showed a noteworthy difference between patients with SR and POAF in the LAA-amputation cohort (Composite endpoint: age < 70 years p = 0.25; > 70 years p = 0.89; CHA_2_DS_2_-VASc-score < 3 p = 0.53; > 3 p = 0.62).

Additionally, in the control groups, regarding the subgroups with an assumed low risk by age (< 70 years) or CHA_2_DS_2_-VASc-Sore (< 3), no significant difference could be observed (composite endpoint: age < 70 years p = 0.58, CHA_2_DS_2_-VASc-score < 3 p = 0.78).

Contrary to that, a significant higher occurrence of the composite endpoint (18.4% vs. 35.2% HR 2.3 95% CI [1.18; 4.50] p = 0.015), driven by a higher rate of all-cause mortality (HR 3.1 95% CI [1.28; 7.32] p = 0.012) and rehospitalization (HR 5.4 95% CI [1.09; 26.97] p = 0.039) but no difference in the stroke rate (p = 0.57) could be detected in elderly patients (> 70 years) with POAF (Table 5).

**Table 5:** Subgroup analysis of older age (> 70 years) and high stroke risk (CHA_2_DS_2_-VASc-socre ≥ 3) in the LAA-amputation group and the control group

	LAA-amputation				Control group			
	SR [n (%)]	POAF [n (%)]	HR [95% CI]	p value	SR [n (%)]	POAF [n (%)]	HR [95% CI]	p value
Age								
Age ≤ 70	84	26			75	27		
Composite endpoint	17 (20.2)	7 (26.9)	1.7 [0.69; 4.26]	0.25	12 (16.0)	7 (25.9)	1.3 [0.50; 3.42]	0.58
All-cause mortality	7 (8.3)	3 (11.5)	1.6 [0.39; 6.35]	0.52	5 (6.7)	3 (11.1)	1.4 [0.32; 5.76]	0.67
Stroke	2 (2.4)	2 (7.7)	3.2 [0.44; 22.49]	0.25	3 (4.0)	2 (7.4)	1.6 [0.25; 10.03]	0.63
Rehospitalization	9 (10.7)	4 (15.4)	1.8 [0.52; 6.06]	0.36	5 (6.7)	3 (11.1)	1.6 [0.38; 7.03]	0.51
Age > 70	84	49			87	54		
Composite endpoint	22 (26.2)	13 (26.5)	1.0 [0.47; 1.90]	0.89	16 (18.4)	19 (35.2)	2.3 [1.18; 4.50]	**0.015**
All-cause mortality	17 (20.2)	12 (24.5)	1.1 [0.53; 2.43]	0.75	8 (9.2)	14 (25.9)	3.1 [1.28; 7.32]	**0.012**
Stroke	2 (2.4)	1 (2.0)	0.7 [0.07; 8.13]	0.80	7 (8.1)	5 (9.3)	1.4 [0.44; 4.47]	0.57
Rehospitalization	3 (3.6)	2 (4.1)	1.0 [0.15; 6.06]	0.96	2 (2.3)	6 (11.1)	5.4 [1.09; 26.97]	**0.039**
Stroke risk								
CHA_2_DS_2_-VASc-score < 3	53	29			68	26		
Composite endpoint	5 (9.4)	4 (13.8)	1.5 [0.41; 5.84]	0.53	10 (14.7)	4 (15.4)	0.8 [0.25; 2.77]	0.76
All-cause mortality	1 (1.9)	3 (10.3)	5.0 [0.5; 48.77]	0.17	3 (4.4)	3 (11.5)	2.4 [0.48; 12.4]	0.29
Stroke	1 (1.9)	1 (3.5)	2.1 [0.13; 34.73]	0.59	3 (4.4)	0 (0.0)	< 0.1 [0; 1007.26]	0.50
Rehospitalization	3 (5.7)	1 (3.5)	0.8 [0.08; 7.75]	0.84	4 (5.9)	1 (3.9)	0.5 [0.06; 5.03]	0.59
CHA_2_DS_2_-VASc-score ≥ 3	115	46			94	55		
Composite endpoint	34 (29.6)	16 (34.8)	1.2 [0.63; 2.15]	0.62	18 (19.2)	22 (40.0)	2.4 [1.27; 4.49]	**0.007**
All-cause mortality	23 (20.0)	12 (26.1)	1.2 [0.57; 2.46]	0.65	10 (10.6)	14 (25.5)	2.5 [1.10; 5.66]	**0.028**
Stroke	3 (2.6)	2 (4.4)	1.7 [0.28; 10.53]	0.56	7 (7.5)	7 (12.7)	1.9 [0.66; 5.57]	0.24
Rehospitalization	9 (7.8)	5 (10.9)	1.4 [0.45; 4.31]	0.56	3 (3.2)	8 (14.6)	5.2 [1.38; 19.81]	**0.015**

*HR* hazard ratio, *POAF* perioperative atrial fibrillation, *SR* sinus rhythm

Bold value indicates *p*-values < 0.05 were considered statistically significant

In concordance to elderly patients, patients with a high CHA_2_DS_2_-VASc-score (> 3) and POAF presented a higher occurrence of the composite endpoint (19.2% vs. 40.0% HR 2.4 95% CI [1.27; 4.49] p = 0.007) with a higher percentage of all-cause mortality (HR 2.5 95% CI [1.10; 5.66] p = 0.028) and rehospitalization (HR 5.2 95% CI [1.38; 19.81] p = 0.015) but no difference in the stroke rate (p = 0.24).

**Fig. 4:**
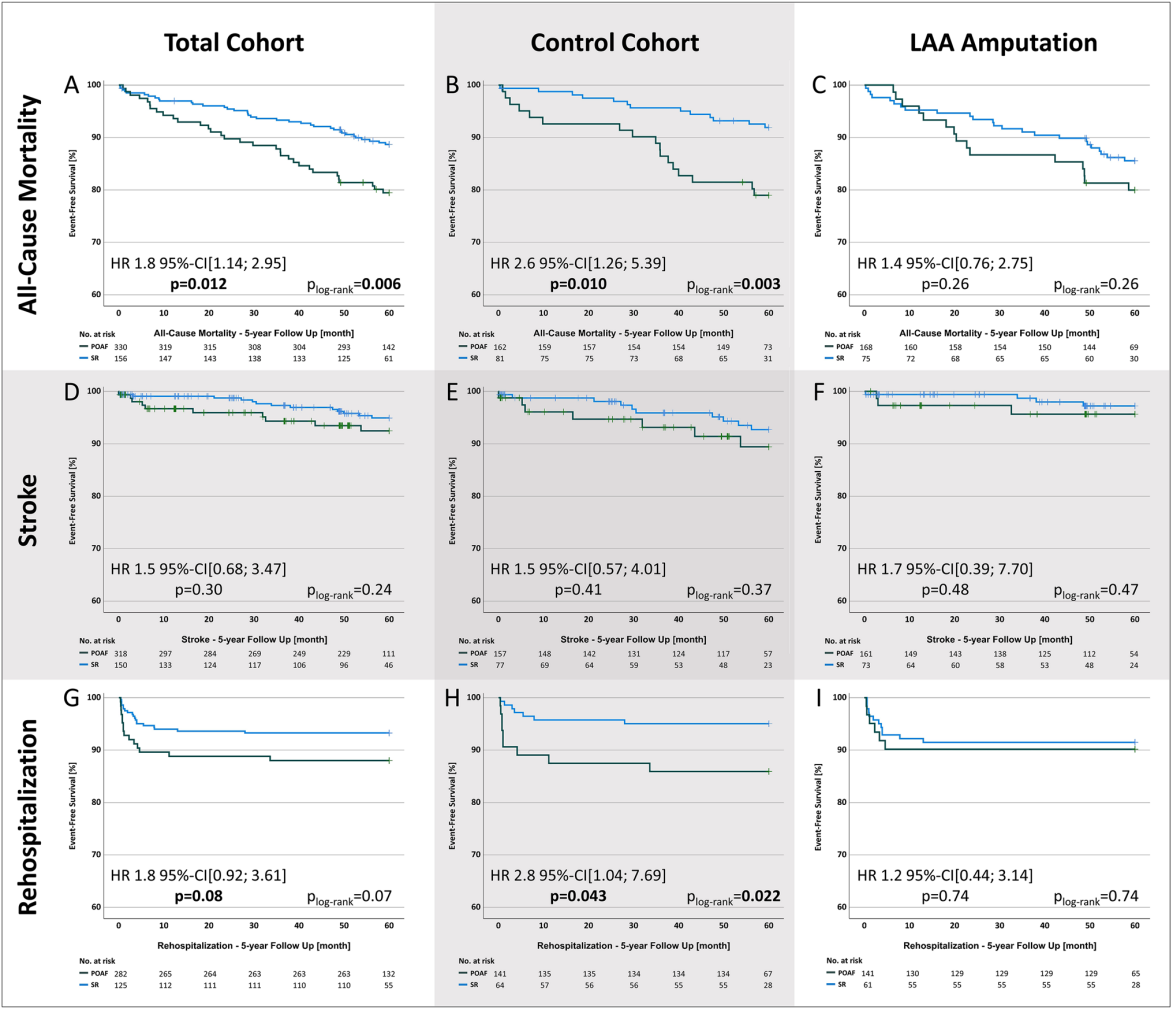
Survival analysis of the secondary endpoints; HR, hazard ratio, POAF, perioperative atrial fibrillation, SR, sinus rhythm

Detailed results are provided in Table 5 and Fig. 4.

## Discussion

This is the first study to assess the long-term impact of LAA-amputation on the outcome in patients with new-onset POAF. Our analysis underlines the considerably worse outcome of patients developing POAF after OPCAB with regard to the composite endpoint of all-cause mortality, stroke, and rehospitalization. Furthermore, the outcome of patients with POAF who underwent LAA-amputation is not inferior compared to patients with maintained SR.

The rising evidence of stroke prevention by LAA-amputation in patients with atrial fibrillation (LAAOS III trial [13]) has led to an essential guideline recommendation for concomitant LAA-amputation [18]. Additionally, stroke prevention in patients with sinus rhythm undergoing cardiac surgery has also been demonstrated [14]. The results of this study go one step further and show that the prognosis of patients with POAF might be restored to that with maintained sinus rhythm. Furthermore, the safety of LAA-amputation with no extension of the cardiopulmonary bypass time and no increase of postoperative complications including bleeding, accompanied by a fast learning curve [19], strengthens the idea of expanding the indication for LAA-amputation in patients with risk factors assessed by the CHA_2_DS_2_-VASC-score. Even concerns about morphological effects on the left atrial geometry such as an increased intraatrial pressure causing dilation of the left atrium resulting in a higher rate of POAF [20], have been resolved [15].

Nevertheless, the conclusive result of this manuscript is the impaired outcome regarding all-cause mortality of patients who developed new-onset POAF after OPCAB surgery. This underlines the severity of an allegedly easy-to-handle postoperative complication and its influence on long-term patient prognosis. The impaired outcome of patients with POAF is in concordance with several studies demonstrating similarly severe mortality [21, 22]. Therefore, it is of utmost interest to identify risk factors for POAF and thus to develop strategies to avoid them.

In the current analysis, patients with POAF and no LAA-amputation showed an impaired outcome in relation to the composite endpoint, driven by all-cause mortality and rehospitalization. In contrast, POAF patients with LAA-amputation had no adverse effects compared to patients with maintained SR. On the other hand, there are hints that patients with maintained SR who received concomitant LAA-amputation have a slightly higher rate of all-cause mortality and rehospitalization without reaching statistical significance (Supplementary Table 2). A prolongation of hospitalization and an onset of heart failure symptoms have been described in literature [23]. In this regard, analysis of the often-discussed humoral impact of the LAA being the main source of atrial natriuretic peptide regulating renal clearance of electrolytes and water [24], is of utmost interest, and further research on this topic is warranted.

Additionally, in contrast to our results, Melduni et al. demonstrated no benefit regarding all-cause mortality and rehospitalization in patients undergoing concomitant LAA-amputation [20]. Hence, each case should be individually discussed, and the decision determined after careful risk factor evaluation for POAF.

Our analysis could proof the beneficial effect with regard to all-cause mortality and rehospitalization, particularly in patients with older age and an elevated stroke/comorbidity risk assessed by the CHA_2_DS_2_VASc-score, which itself includes older age. Thus, taking the current results into account, the CHA_2_DS_2_VASc-score, which is already being the main decision-making variable [6, 18], should be used for patients without preoperative AF, as well.

Patient selection has to always be a core point in prophylactic treatment approaches such as LAA-amputation, since the weight of responsibility is even greater, when recommending an additional procedure for treatment of a potential postoperative complication that could increase the risk of adverse outcomes, even though it might not manifest itself in the first place. When discussing a broad prophylactic recommendation, it is of utmost interest to establish the best possible technique for LAA amputation, which is still depending mostly on established habits, institutional experience, and economic considerations with the most commonly employed techniques of suturing, stapling and clipping [25]. This necessitates a levelheaded evidence-based discussion on general technical differences, patient-based differences and surgery-based differences (e.g. minimally invasive cardiac surgery, OPCAB, conventional cardiac surgery) to attain the required knowledge to form the surgeon’s preference, which is and will remain the decisive factor in making the choice.

Interestingly, in terms of the single endpoint stroke, no notable difference could be revealed. This raises the question of whether some stroke events are concealed within all-cause mortality without further investigation of the mortality cause. However, since several studies’ reporting’s range from “association” [3, 26, 27] to “no association”, the impact of POAF on long-term stroke risk needs to be further elucidated. Hsu et al. were able to demonstrate similar outcomes after POAF in a cohort of over 8000 patients with a higher mortality and rehospitalization rate but no increase in the stroke rate [28]. In contrast, sub-results of the EXCEL trial showed comparable results for patients with POAF with poor outcomes, indicating that POAF could be identified as an independent risk factor for stroke [29].

### Limitations

Several limitations apply to our study. The main limitation of the analysis is the retrospective and single center study design. Furthermore, the anticoagulation strategy and patients’ compliance were not part of the follow-up, so a potential bias due to medical treatment differences cannot be ruled out. Nonetheless, similar to the LAAOS III cohorts [13] and following the guideline recommendations [6], all patients were treated with oral anticoagulation at least during hospitalization. Detailed information regarding LAA-amputation strategy was not given, however, the cohort assessed is an amputation-only cohort.

## Conclusion

POAF is associated with a higher rate of the composite endpoint of all-cause mortality, stroke and rehospitalization. The combined endpoint in patients with LAA-amputation concomitant with OPCAB surgery developing new-onset POAF in a 5-year follow-up was not increased compared to a control cohort maintaining sinus rhythm.

### Supplementary Information

Below is the link to the electronic supplementary material.

Supplementary file1 (DOCX 46 kb)

## Data Availability

The data underlying this article will be shared upon reasonable request to the corresponding author.

## Abbreviations

95% CI: 95% Confidence interval
AF: Atrial fibrillation
ASA: American Society of Anesthesiologists
BMI: Body mass index
CAD: Coronary artery disease
CCS: Canadian Cardiovascular Society
CHA_2_DS_2_VASc: Congestive heart failure, hypertension, age > 75 (doubled), diabetes, stroke (doubled), vascular disease, age 65 to 74 and sex category (female)
CPR: Cardiopulmonary resuscitation
CRT: Controlled randomized trial
DVT: Deep vein thrombosis
ECLS: Extracorporeal life support
EF: Ejection fraction
HR: Hazard ratio
IABP: Intra-aortic balloon pump
IQR: Interquartile range
LAA: Left atrial appendage
NYHA: New York Heart Association
OPCAB: Off-pump coronary artery bypass grafting
OR: Odds ratio
PAPs: Systolic pulmonary arterial pressure
POAF: Perioperative atrial fibrillation
PS: Propensity score
SD: Standard deviation
SR: Sinus rhythm
STROBE: STrengthening the Reporting of OBservational studies in Epidemiology
TSH: Thyroid stimulating hormone
VT: Ventricular tachycardia

## References

[1] Kaw R, Hernandez AV, Masood I, Gillinov AM, Saliba W, Blackstone EH (2011). Short- and long-term mortality associated with new-onset atrial fibrillation after coronary artery bypass grafting: a systematic review and meta-analysis. J Thorac Cardiovasc Surg.

[2] Almassi GH, Wagner TH, Carr B, Hattler B, Collins JF, Quin JA (2015). Postoperative atrial fibrillation impacts on costs and one-year clinical outcomes: the Veterans Affairs Randomized On/Off Bypass Trial. Ann Thorac Surg.

[3] Thoren E, Wernroth ML, Christersson C, Grinnemo KH, Jideus L, Stahle E (2020). Compared with matched controls, patients with postoperative atrial fibrillation (POAF) have increased long-term AF after CABG, and POAF is further associated with increased ischemic stroke, heart failure and mortality even after adjustment for AF. Clin Res Cardiol.

[4] Echahidi N, Pibarot P, O'Hara G, Mathieu P (2008). Mechanisms, prevention, and treatment of atrial fibrillation after cardiac surgery. J Am Coll Cardiol.

[5] Gillinov AM, Bagiella E, Moskowitz AJ, Raiten JM, Groh MA, Bowdish ME (2016). Rate control versus rhythm control for atrial fibrillation after cardiac surgery. N Engl J Med.

[6] Hindricks G, Potpara T, Dagres N, Arbelo E, Bax JJ, Blomström-Lundqvist C (2021). 2020 ESC Guidelines for the diagnosis and management of atrial fibrillation developed in collaboration with the European Association for Cardio-Thoracic Surgery (EACTS): the Task Force for the diagnosis and management of atrial fibrillation of the European Society of Cardiology (ESC) Developed with the special contribution of the European Heart Rhythm Association (EHRA) of the ESC. Eur Heart J.

[7] Chiang CE, Naditch-Brule L, Murin J, Goethals M, Inoue H, O'Neill J (2012). Distribution and risk profile of paroxysmal, persistent, and permanent atrial fibrillation in routine clinical practice: insight from the real-life global survey evaluating patients with atrial fibrillation international registry. Circ Arrhythm Electrophysiol.

[8] Chugh SS, Havmoeller R, Narayanan K, Singh D, Rienstra M, Benjamin EJ (2014). Worldwide epidemiology of atrial fibrillation: a Global Burden of Disease 2010 Study. Circulation.

[9] Oldgren J, Healey JS, Ezekowitz M, Commerford P, Avezum A, Pais P (2014). Variations in cause and management of atrial fibrillation in a prospective registry of 15,400 emergency department patients in 46 countries: the RE-LY Atrial Fibrillation Registry. Circulation.

[10] Blackshear JL, Odell JA (1996). Appendage obliteration to reduce stroke in cardiac surgical patients with atrial fibrillation. Ann Thorac Surg.

[11] Damiano RJ (2008). What is the best way to surgically eliminate the left atrial appendage?. J Am Coll Cardiol.

[12] Ramlawi B, Abu Saleh WK, Edgerton J (2015). The left atrial appendage: target for stroke reduction in atrial fibrillation. Methodist Debakey Cardiovasc J.

[13] Whitlock RP, Belley-Cote EP, Paparella D, Healey JS, Brady K, Sharma M (2021). Left atrial appendage occlusion during cardiac surgery to prevent stroke. N Engl J Med.

[14] Gercek M, Borgermann J, Gercek M, Gummert J (2023). Left atrial appendage amputation concomitant to cardiac surgery in patients with sinus rhythm. Eur J Cardiothorac Surg.

[15] Gercek M, Ghabrial M, Glaubitz L, Kuss O, Aboud A, Paluszkiewicz L (2021). Impact of left atrial appendage amputation on left atrial morphology and rhythm after off-pump CABG. Thorac Cardiovasc Surg.

[16] Austin PC (2014). A comparison of 12 algorithms for matching on the propensity score. Stat Med.

[17] Kuss O (2013). The z-difference can be used to measure covariate balance in matched propensity score analyses. J Clin Epidemiol.

[18] Vahanian A, Beyersdorf F, Praz F, Milojevic M, Baldus S, Bauersachs J (2022). 2021 ESC/EACTS Guidelines for the management of valvular heart disease. Eur Heart J.

[19] Healey JS, Crystal E, Lamy A, Teoh K, Semelhago L, Hohnloser SH (2005). Left Atrial Appendage Occlusion Study (LAAOS): results of a randomized controlled pilot study of left atrial appendage occlusion during coronary bypass surgery in patients at risk for stroke. Am Heart J.

[20] Melduni RM, Schaff HV, Lee HC, Gersh BJ, Noseworthy PA, Bailey KR (2017). Impact of left atrial appendage closure during cardiac surgery on the occurrence of early postoperative atrial fibrillation, stroke, and mortality: a propensity score-matched analysis of 10,633 patients. Circulation.

[21] El-Chami MF, Kilgo P, Thourani V, Lattouf OM, Delurgio DB, Guyton RA (2010). New-onset atrial fibrillation predicts long-term mortality after coronary artery bypass graft. J Am Coll Cardiol.

[22] Phan K, Ha HS, Phan S, Medi C, Thomas SP, Yan TD (2015). New-onset atrial fibrillation following coronary bypass surgery predicts long-term mortality: a systematic review and meta-analysis. Eur J Cardiothorac Surg.

[23] Mahmood E, Matyal R, Mahmood F, Xu X, Sharkey A, Chaudhary O (2020). Impact of left atrial appendage exclusion on short-term outcomes in isolated coronary artery bypass graft surgery. Circulation.

[24] Lakkireddy D, Turagam M, Afzal MR, Rajasingh J, Atkins D, Dawn B (2018). Left atrial appendage closure and systemic homeostasis: the LAA HOMEOSTASIS study. J Am Coll Cardiol.

[25] Salzberg SP, Emmert MY, Caliskan E (2017). Surgical techniques for left atrial appendage exclusion. Herzschrittmacherther Elektrophysiol.

[26] Ahlsson A, Fengsrud E, Bodin L, Englund A (2010). Postoperative atrial fibrillation in patients undergoing aortocoronary bypass surgery carries an eightfold risk of future atrial fibrillation and a doubled cardiovascular mortality. Eur J Cardiothorac Surg.

[27] Gialdini G, Nearing K, Bhave PD, Bonuccelli U, Iadecola C, Healey JS (2014). Perioperative atrial fibrillation and the long-term risk of ischemic stroke. JAMA.

[28] Hsu JC, Huang CY, Chuang SL, Yu HY, Chen YS, Wang CH (2021). Long term outcome of postoperative atrial fibrillation after cardiac surgery—a propensity score-matched cohort analysis. Front Cardiovasc Med.

[29] Kosmidou I, Chen S, Kappetein AP, Serruys PW, Gersh BJ, Puskas JD (2018). New-onset atrial fibrillation after PCI or CABG for left main disease: the EXCEL trial. J Am Coll Cardiol.

