# Supplementary material for: Five-year-outcome of new-onset perioperative atrial fibrillation after left atrial appendage amputation concomitant with cardiac surgery
Source: Clin Res Cardiol. 2023 Jul 10;112(12):1800–11. doi: 10.1007/s00392-023-02255-8 (PMC10698101; doi:10.1007/s00392-023-02255-8)
Supplement: Supplementary file 1 — Supplementary file1 (DOCX 46 kb) [file 392_2023_2255_MOESM1_ESM.docx]

## Supplementary Table 1 Baseline characteristics by development of POAF in the PS matched cohorts

| **Variable** | **PS Matched Cohorts** | | | |
| --- | --- | --- | --- | --- |
|  | **POAF** | | **OR 95%CI** | **p-value** |
|  | **No (n=330)** | **Yes (n=156)** |  |  |
| **NYHA (%)** | 2.0±0.8 | 2.1±0.8 | -0.27; 0.03 | 0.13 |
| **NYHA I** | 98 (29.7) | 42 (26.9) |  |  |
| **NYHA II** | 147 (44.6) | 61 (39.1) |  |  |
| **NYHA III** | 82 (24.9) | 51 (32.7) |  |  |
| **NYHA IV** | 3 (0.9) | 2 (1.3) |  |  |
| **Ejection Fraction (SD)** | 58.1±9.6 | 57.0±10.8 | -0.85; 2.98 | 0.27 |
| **DVT (%)** | 6 (1.8) | 3 (1.9) | 1.1 [0.26; 4.29] | 0.94 |
| **Beta-Blocker (%)** | 221 (67.0) | 115 (73.7) | 1.4 [0.91; 2.11] | 0.13 |
| **Calcium Antagonist (%)** | 75 (22.7) | 48 (30.8) | 1.1 [0.99; 2.31] | 0.06 |
| **EuroSCORE2 (IQR)** | 1.7±1.5 | 2.1±2.6 | -0.69; 0.05 | 0.10 |
| **CHA_2_DS_2_-VASc-Score (SD)** | 3.0±1.3 | 3.2±1.4 | -0.38; 0.13 | 0.34 |
| **Age (SD)** | 68.7±8.0 | 70.9±7.1 | -3.66; -0.70 | **<0.01** |
| **BMI (SD)** | 28.9±4.2 | 29.2±4.5 | -1.07; 0.57 | 0.56 |
| **TSH [µU/ml]. (IQR)** | 1.3±1.1 | 1.2±1.0 | -0.07; 0.34 | 0.18 |
| **ASA Classification (%)** | 2.8±0.6 | 2.9±0.5 | -0.23; -0.01 | **0.03** |
| **normal healthy patient** | 21 (6.4) | 4 (2.7) |  |  |
| **patient with mild systemic disease** | 28 (8.5) | 12 (7.7) |  |  |
| **patient with severe systemic disease** | 268 (81.2) | 129 (82.7) |  |  |
| **patient with severe systemic disease that is a constant threat to life** | 13 (3.9) | 11 (7.1) |  |  |
| **CCS (%)** | 2.3±1.1 | 2.4±1.1 | -0.33; 0.08 | 0.26 |
| **CCS I** | 112 (33.9) | 48 (30.8) |  |  |
| **CCS II** | 72 (21.8) | 39 (25.0) |  |  |
| **CCS III** | 48 (14.6) | 23 (14.7) |  |  |
| **CCS IV** | 4 (1.2) | 5 (3.2) |  |  |
| **Female (%)** | 74 (22.4) | 26 (16.7) | 0.7 [0.42; 1.13] | 0.14 |
| **Myocardial Infarction (%)** | 65 (19.7) | 32 (20.5) | 1.1 [0.65; 1.69] | 0.83 |
| **0 to 6 hours** | 0 (0) | 1 (0.6) |  |  |
| **6 to 24 hours** | 1 (0.3) | 0 (0) |  |  |
| **24 to 48 hours** | 3 (0.9) | 3 (1.9) |  |  |
| **48 hours to 7 days** | 3 (0.9) | 2 (1.3) |  |  |
| **7 to 21 days** | 12 (3.6) | 3 (1.9) |  |  |
| **21 to 90 days** | 7 (2.1) | 4 (2.6) |  |  |
| **longer than 90 days** | 39 (11.8) | 19 (12.2) |  |  |
| **Arterial Hypertension (%)** | 294 (89.1) | 139 (89.1) | 1.0 [0.54; 1.84] | >0.99 |
| **Diabetes Mellitus Type 2 (%)** | 164 (49.7) | 60 (38.5) | 0.6 [0.43; 0.93] | **0.020** |
| **Pulmonary Disease (%)** | 20 (6.1) | 14 (9.0) | 1.5 [0.75; 3.11] | 0.24 |
| **Hyperlipidemia (%)** | 310 (93.9) | 146 (93.6) | 0.9 [0.43; 2.06] | 0.88 |
| **Smoking (%)** | 121 (36.7) | 67 (43.0) | 1.3 [0.88; 1.92] | 0.18 |
| **Coronary Artery Disase (%)** | 2.8±0.4 | 2.9±0.4 | -0.15; 0.01 | 0.09 |
| **1-CAD** | 6 (1.8) | 3 (1.9) |  |  |
| **2-CAD** | 50 (15.2) | 13 (8.3) |  |  |
| **3-CAD** | 274 (83.0) | 140 (89.7) |  |  |
| **OP time [min]** | 198.3±43.4 | 197.2±40.2 | -6.99; 9.19 | 0.79 |
| **Potassium [mmol/l]** | 4.02±0.41 | 4.06±0.41 | -0.12; 0.04 | 0.39 |
| **Sodium [mmol/l]** | 139.3±2.9 | 139.3±3.3 | -0.54; 0.61 | 0.90 |
| **Calcium [mmol/l]** | 2.4±0.1 | 2.4±0.1 | -0.02; 0.02 | 0.84 |
| **Creatinine [mg/dl]** | 1.1±0.8 | 1.1±0.5 | -0.09; 0.20 | 0.40 |

Abbreviations: ASA, American Society of Anesthesiologists; BMI, body mass index; CAD, coronary artery disease; DVT, deep vein thrombosis; IQR, interquartile range; NYHA, New York Heart Association; TSH, thyroid stimulating hormone.

## Supplementary Table 2 Five-Year Results of the primary composite and single endpoints in patients and subgroups of patients with perioperative maintenance of sinus rhythm and perioperative atrial fibrillation in the LAA-amputation group and the control group.

|  | | **Sinus Rhythm** | | | | **POAF** | | | |
| --- | --- | --- | --- | --- | --- | --- | --- | --- | --- |
|  |  | **Control**  **[n (%)]** | **LAA Amp.**  **[n (%)]** | **HR [95%CI]** | **p-value** | **Control**  **[n (%)]** | **LAA Amp.**  **[n (%)]** | **HR [95%CI]** | **p-value** |
| **All** | **All Patients** | 162 | 168 |  |  | 81 | 75 |  |  |
|  | Composite Endpoint | 28 (17.3) | 39 (23.2) | 1.4 [0.88; 2.33] | 0.15 | 26 (32.1) | 20 (26.7) | 0.8 [0.42; 1.38] | 0.37 |
|  | All-Cause Mortality | 13 (8.0) | 24 (14.3) | 1.8 [0.92; 3.57] | 0.08 | 17 (21.0) | 15 (20.0) | 0.9 [0.44; 1.81] | 0.76 |
|  | Stroke | 10 (6.2) | 4 (2.4) | 0.4 [0.13; 1.34] | 0.14 | 7 (8.6) | 3 (4.0) | 0.4 [0.10; 1.53] | 0.18 |
|  | Rehospitalization | 7 (4.3) | 12 (7.1) | 1.7 [0.67; 4.35] | 0.26 | 9 (11.1) | 6 (8.0) | 0.7 [0.24; 1.91] | 0.46 |
| **Age** | **Age ≤ 70** | 82 | 92 |  |  | 31 | 30 |  |  |
|  | Composite Endpoint | 13 (15.9) | 18 (19.6) | 1.2 [0.59; 2.46] | 0.62 | 10 (32.3) | 7 (23.3) | 0.7 [0.28; 1.96] | 0.55 |
|  | All-Cause Mortality | 6 (7.3) | 8 (8.7) | 1.1 [0.37; 3.08] | 0.91 | 6 (19.4) | 3 (10.0) | 0.6 [0.14; 2.24] | 0.41 |
|  | Stroke | 3 (3.7) | 2 (2.2) | 0.5 [0.09; 3.23] | 0.49 | 3 (9.7) | 2 (6.7) | 0.6 [0.10; 3.65] | 0.58 |
|  | Rehospitalization | 5 (6.1) | 9 (9.8) | 1.6 [0.54; 4.88] | 0.39 | 4 (12.9) | 4 (13.3) | 1.2 [0.28; 5.13] | 0.81 |
|  | **Age >70** | 80 | 76 |  |  | 50 | 45 |  |  |
|  | Composite Endpoint | 15 (18.8) | 21 (27.6) | 1.7 [0.88; 3.36] | 0.11 | 16 (32.0) | 13 (28.9) | 0.7 [0.33; 1.57] | 0.41 |
|  | All-Cause Mortality | 7 (8.8) | 16 (21.1) | 2.6 [1.08; 6.39] | **0.034** | 11 (22.0) | 12 (26.7) | 1.1 [0.44; 2.52] | 0.91 |
|  | Stroke | 7 (8.8) | 2 (2.6) | 0.4 [0.09; 2.14] | 0.31 | 4 (8.0) | 1 (2.2) | 0.2 [0.03; 2.15] | 0.20 |
|  | Rehospitalization | 2 (2.5) | 3 (4.0) | 1.6 [0.27; 9.72] | 0.60 | 5 (10.0) | 2 (4.4) | 0.4 [0.08; 2.24] | 0.31 |
| **Stroke Risk** | **CHA_2_DS_2_-VASc-Score < 3** | 68 | 53 |  |  | 26 | 29 |  |  |
|  | Composite Endpoint | 10 (14.7) | 5 (9.4) | 0.6 [0.21; 1.84] | 0.39 | 4 (15.4) | 4 (13.8) | 0.9 [0.22; 3.48] | 0.84 |
|  | All-Cause Mortality | 3 (4.4) | 1 (1.9) | 0.5 [0.05; 4.51] | 0.51 | 3 (11.5) | 3 (10.3) | 0.9 [0.18; 4.44] | 0.89 |
|  | Stroke | 3 (4.4) | 1 (1.9) | 0.5 [0.05; 4.34] | 0.49 | 0 (0) | 1 (3.5) | 53.2 [0.00; 59.55*10^8^] | 0.63 |
|  | Rehospitalization | 4 (5.9) | 3 (5.7) | 0.8 [0.18; 3.74] | 0.79 | 1 (3.9) | 1 (3.5) | 1.2 [0.07; 18.59] | 0.92 |
|  | **CHA_2_DS_2_-VASc-Score ≥ 3** | 94 | 115 |  |  | 55 | 46 |  |  |
|  | Composite Endpoint | 18 (19.2) | 34 (29.6) | 1.7 [0.95; 3.05] | 0.07 | 22 (40.0) | 16 (34.8) | 0.8 [0.38; 1.48] | 0.41 |
|  | All-Cause Mortality | 10 (10.6) | 23 (20.0) | 2.0 [0.93; 4.19] | 0.07 | 14 (25.5) | 12 (26.1) | 0.8 [0.37; 1.92] | 0.68 |
|  | Stroke | 7 (7.5) | 3 (2.6) | 0.4 [0.10; 1.53] | 0.17 | 7 (12.7) | 2 (4.4) | 0.3 [0.07; 1.59] | 0.17 |
|  | Rehospitalization | 3 (3.2) | 9 (7.8) | 2.6 [0.69; 9.52] | 0.16 | 8 (14.6) | 5 (10.9) | 0.7 [0.22; 2.12] | 0.51 |

Abbreviations: HR, hazard ratio; POAF, perioperative atrial fibrillation; SR, sinus rhythm.
